# Supplementary material for: Impact of the Electric Vehicle Policies on Environment and Health in the Beijing–Tianjin–Hebei Region
Source: Int J Environ Res Public Health. 2021 Jan 13;18(2):623. doi: 10.3390/ijerph18020623 (PMC7828382; doi:10.3390/ijerph18020623)
Supplement: Supplementary file 1 [file ijerph-18-00623-s001.pdf]

Impact of the electric vehicle policies on environment and health in the Beijing-Tianjin-Hebei Region

Table A1. The automobile ownership Projection

Unit: million		Total	PC	LC	MT	HT	HB
Beijing	2010	4.74	4.33	0.28	0.04	0.04	0.05
	2015	5.54	5.02	0.36	0.03	0.07	0.06
	2020	7.45	6.99	0.31	0.02	0.06	0.06
	2025	7.45	7.04	0.21	0.01	0.13	0.05
	2030	6.69	6.32	0.12	0.01	0.21	0.04
Tianjin	2010	1.70	1.43	0.19	0.02	0.04	0.02
	2015	3.17	2.78	0.28	0.01	0.06	0.03
	2020	4.11	3.57	0.34	0.01	0.16	0.03
	2025	4.36	3.71	0.33	0.00	0.27	0.05
	2030	3.89	3.37	0.08	0.00	0.37	0.07
Hebei	2010	5.18	3.76	0.80	0.12	0.46	0.05
	2015	10.76	9.17	0.99	0.05	0.50	0.05
	2020	17.68	15.69	1.15	0.57	0.20	0.06
	2025	25.14	22.96	1.04	0.66	0.44	0.04
	2030	30.48	27.81	0.79	0.90	0.98	0.01

Table A2. National electric vehicles' proportion of total civil automobile ownership[2]

Proportion (%)	Passenger cars		Trucks		Buses	
	EV	PHEV	EV	PHEV	EV	PHEV
2010	0	0	0	0	0	0
2015	0.25	0.01	0	0	0.5	0
2020	2	0.03	0	0	7.8	0
2025	8	0.05	0	1	11.5	0.5
2030	20	1	0	5	18	2

[2] We have slightly changed the result from (Peng T, et.al, 2018)[23] based on the total ownership forecast of EVs (China Automotive Technology Research Center, 2016) [47].

Table A3. The ownership projection of electric vehicles

Region	Unit: million	EV			PHEV		
		PC	Truck	Bus	PC	Truck	Bus
Beijing	2015	0.01	0.00	0.01	0.00	0.00	0.00
	2020	0.21	0.00	0.03	0.00	0.00	0.00
	2025	0.99	0.00	0.03	0.15	0.01	0.00
	2030	2.53	0.00	0.04	0.82	0.14	0.01
Tianjin	2015	0.01	0.00	0.00	0.00	0.00	0.00
	2020	0.14	0.00	0.01	0.01	0.00	0.00
	2025	0.59	0.00	0.02	0.15	0.02	0.00
	2030	1.59	0.00	0.02	0.51	0.21	0.00
Hebei	2015	0.02	0.00	0.01	0.00	0.00	0.00
	2020	0.42	0.00	0.02	0.04	0.00	0.00

2025	2.07	0.00	0.04	0.09	0.02	0.00
2030	5.56	0.00	0.05	0.83	0.12	0.00

Table A4. (a) The fossil energy consumption projection

Unit:	Fossil-Energy-Consumption Vehicle					PHEV		
L/100km	PC	LC	MT	HT	HB	PC	Bus	Truck
2010	8.18	14.54	28.01	44.66	28.64	\	\	\
2015	7.29	12.38	23.91	40.15	25.09	2.21	19.25	12.65
2020	5.00	10.33	18.66	36.18	20.00	4.28	9.81	6.57
2025	4.00	9.76	17.62	34.17	18.89	3.85	8.83	5.91
2030	3.20	9.19	16.58	32.16	17.78	3.43	7.85	5.26

Table A4. (b) The electricity consumption projection

Unit:	Electric Vehicle			PHEV		
kWh/100km	PC	Bus	Truck	PC	Bus	Truck
2010	\	\	\	\	\	\
2015	15.39	45.48	29.89	21.51	49.67	32.64
2020	12.00	35.00	35.00	12.00	35.00	35.00
2025	10.80	32.00	32.00	10.80	32.00	32.00
2030	9.72	30.00	30.00	9.72	30.00	30.00

Table A5. The energy consumption and generation

Unit:	Consumption			Total Generation			Thermal Power			Input
Billion kWh	Beijing	Tianjin	Hebei	Beijing	Tianjin	Hebei	Beijing	Tianjin	Hebei	
2010	81.0	64.6	269.2	26.9	58.9	199.3	26.2	56.0	192.6	129.6
2015	95.3	80.1	317.6	42.1	62.3	249.8	41.1	61.4	227.2	138.7
2020	118.3	99.0	418.4	43.0	81.5	319.6	42.3	80.0	295.2	191.7
2025	138.6	118.1	503.1	50.3	96.7	379.4	49.5	94.8	348.3	233.5
2030	158.9	137.2	587.7	57.6	111.9	439.2	56.8	109.6	401.5	275.3

Table A6. Fossil energy consumption projection by civil automobiles

Unit:	FOS			REN			Reduction
PJ	Beijing	Tianjin	Hebei	Beijing	Tianjin	Hebei	
2010	317.29	150.88	748.46	317.09	146.32	723.63	2.4%
2015	339.10	225.45	949.98	321.84	212.68	775.22	13.5%
2020	308.57	266.86	894.68	280.13	259.29	604.07	22.2%
2025	289.98	311.76	1099.40	254.27	283.19	747.40	24.5%
2030	267.01	314.34	1377.15	188.05	247.59	864.39	33.6%

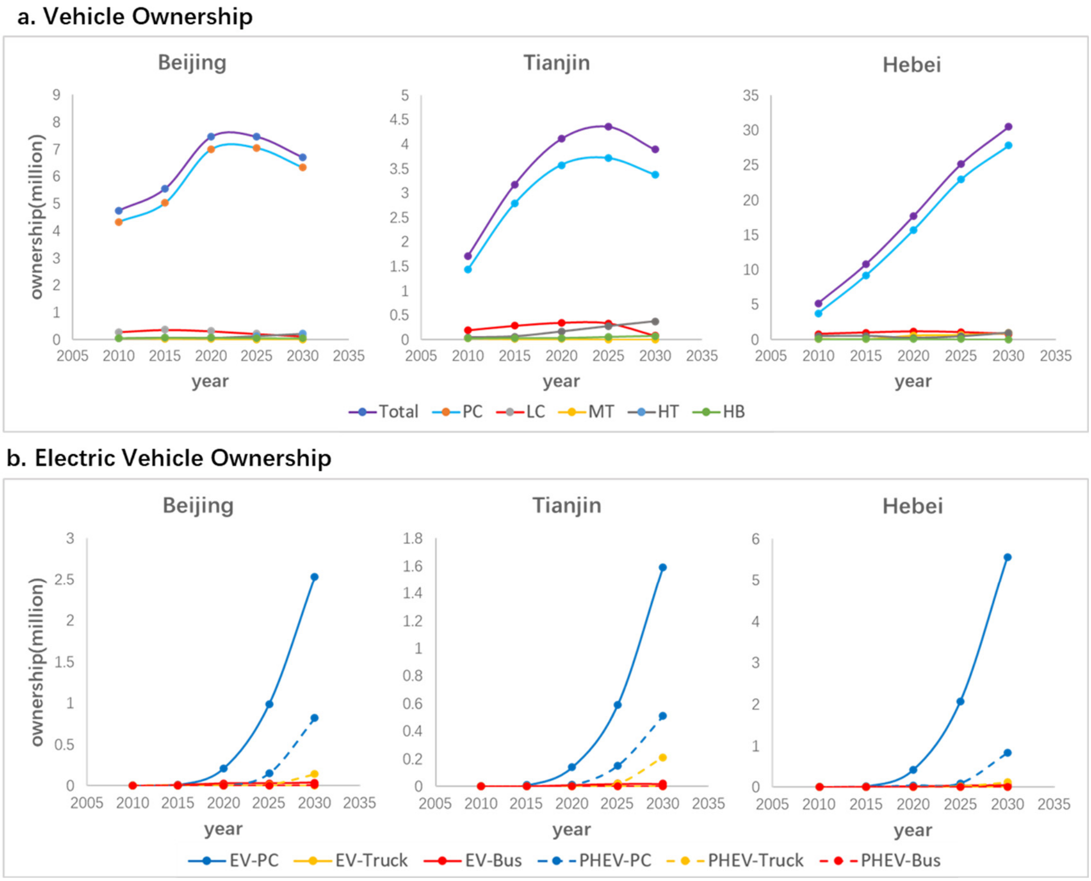

Fig.A1. Vehicle ownership projection
